# 

**Published:** 1884-06

**Authors:** 


### The following Periodicals are received in Exchange with the Bristol Medico-Chirurgical Journal.

### AMERICA.

CANADA.

*Monthly.*

*Canada Medical and Surgical Journal.* Montreal: Gazette Printing Company.

*The Canada Lancet.* Toronto: 303 Church Street.

UNITED STATES.

*Weekly.*

*The Medical and Surgical Reporter.* Philadelphia: 115 South Seventh Street.

*The Medical Record.* New York: William Wood & Co.

*The New York Medical Journal.* New York: D. Appleton & Co.

*Fortnightly.*

*Philadelphia Medical Times.* Philadelphia: J. B. Lippincott & Co.

*Monthly.*

*Index Medicus.* New York: F. Leypoldt.

*The American Practitioner.* Louisville: John P. Morton and Company.

*The Polyclinic.* Philadelphia: P. Blakiston, Son & Co.

*The Saint Louis Medical and Surgical Journal.* Saint Louis: 2622 Washington Avenue.

*The Therapeutic Gazette.* Detroit: George S. Davis.

*The Analectic.* New York: G. P. Putnam's Sons.

*Quarterly.*

*The American Journal of the Medical Sciences.* Philadelphia: Henry C. Lea's Son & Co.

*The American Psychological Journal.* Philadelphia: P. Blakiston, Son & Co.

*The International Review of Medical and Surgical Technics.* Boston: 51 Union Park.

### AUSTRALASIA.

AUSTRALIA.

*Monthly.*

*The Australasian Medical Gazette.* Sydney: L. Bruck.

*The Australian Medical Journal.* Melbourne: Stillwell & Co.

### EUROPE.

BELGIUM.

*Monthly.*

*Bulletin de l'Académie Royale de Médecine de Belgique.* Brussels: A. Manceaux.

BRITISH ISLES.

*Weekly.*

*British Medical Journal.* London: 161A Strand.

*Medical Times and Gazette.* London: J. & A. Churchill.

*The Lancet.* London: 423 Strand.

*The Edinburgh Clinical and Pathological Journal.* Edinburgh: Bell & Bradfute.

*Monthly.*

*The Birmingham Medical Review.* Birmingham: Cornish Bros.

*The London Medical Record.* London: Smith, Elder, & Co.

*The Sanitary Record.* London: Smith, Elder, & Co.

*Edinburgh Medical Journal.* Edinburgh: Oliver and Boyd.

*The Glasgow Medical Journal.* Glasgow: Alex. Macdougall.

*The Dublin Journal of Medical Science.* Dublin: Fannin & Co.

*Quarterly.*

*The Asclepiad.* London: Eade and Caulfield.

*Half-yearly.*

*The Liverpool Medico-Chirurgical Journal.* Liverpool: Medical Institution.

*The Retrospect of Medicine.* London: Simpkin, Marshall, & Co.

*Yearly.*

*The Medical Annual.* London: Henry Kimpton.

FRANCE.

*Daily.*

*Le Journal Médical Quotidien.* Paris: 7 Rue d'Argenson.

*Three times a week.*

*Gazette des Hôpitaux.* Paris: 4 Rue de l'Odéon.

*L'Union Médicale.* Paris: 11 Rue de la Grange-Batelière.

*Weekly.*

*Bulletin de l'Académie de Médecine.* Paris: G. Masson.

*Gazette Médicale de Paris.* Paris: 8 Place de l'Odéon.

*Le Progrès Médical.* Paris: 14 Rue des Carmes.

ITALY.

*Monthly.*

*Lo Sperimentale.* Florence: 35 Via degli Alfani.

### *BOOKS, &c., RECEIVED.*

*The Treatment of Backward Displacements of the Uterus and of Prolapsus Uteri by the New Method of Shortening the Round Ligaments.* By W. Alexander, M.D. London: J. & A. Churchill. 1884.

*An Index of Surgery.* By C. B. Keetley, F.R.C.S. Second edition. London: Smith, Elder, & Co. 1884.

*The Leamington Waters.* By F. W. Smith, M.D. London: H. K. Lewis. 1884.

*Discussion on Albuminuria.* Glasgow Pathological and Clinical Society. Glasgow: Macdougall. 1884.

*Albumen and Sugar Testing.* By Geo. Johnson, M.D. London: Smith, Elder, & Co. 1884.

*Syphilis and Pseudo-Syphilis.* By Alfred Cooper, F.R.C.S. London: J. & A. Churchill. 1884.

### SURGICAL APPLIANCES.

The attention of the Medical Profession is directed to the fact that all kinds of Trusses, Belts, Bandages, and other Surgical Appliances are made by

**J. G. PLUMLEY, Bristol Bridge, BRISTOL.**

A Large Assortment of Elastic Goods, Enemas, Bougies, Catheters, Pessaries, Ice Bags, &c., kept in stock.

*PRIVATE ROOMS FOR FITTING.* *AN EXPERIENCED FEMALE ATTENDANT.*

### Bristol Medico-Chirurgical Journal.

JUNE, 1884.

*Cases for binding, price 7d. each, may be had of the Publisher,*
J. W. ARROWSMITH, *Quay Street, Bristol.*

